# Past Achievements, Present Gaps, and Future Priorities in Pneumocystis jirovecii Research: A Global Bibliometric Analysis

**DOI:** 10.3390/pathogens15050530

**Published:** 2026-05-14

**Authors:** Bryan Ortiz, Jonathan Muñoz-Tabora, Kateryn Aguilar, Gustavo Fontecha, Gabriela Matamoros, Lelany Pineda-Garcia, Nancy Alvarez-Corrales, Jaime Palomares-Marín, Claudia L. Cueto-Aragón, Yaxsier de Armas, Enrique J. Calderón

**Affiliations:** 1 Instituto de Investigaciones en Microbiología, Facultad de Ciencias, Universidad Nacional Autónoma de Honduras, Tegucigalpa 11101, Honduras; bryan.ortiz@unah.edu.hn (B.O.); kateryn.aguilar@unah.edu.hn (K.A.); gustavo.fontecha@unah.edu.hn (G.F.); gabriela.matamoros@unah.edu.hn (G.M.); 2 Electric Engineering Department, National Autonomous University of Honduras, Tegucigalpa 04001, Honduras; jonathan.munoz@unah.edu.hn; 3 Departamento de Microbiología, Escuela de Microbiología, Facultad de Ciencias, Universidad Nacional Autónoma de Honduras, Tegucigalpa 11101, Honduras; lelany.pineda@unah.edu.hn (L.P.-G.); nancy.alvarez@unah.edu.hn (N.A.-C.); 4 Departamento de Microbiología y Patología, Centro Universitario de Ciencias de la Salud, Universidad de Guadalajara, Guadalajara 44100, Mexico; jaime.palomares@academicos.udg.mx; 5 Hospital Infantil “Eva Sámano de López Mateos”, Morelia 58253, Mexico; claudiacueto22@gmail.com; 6 Pathology Department, Hospital Center, Institute of Tropical Medicine “Pedro Kourí”, Havana 11400, Cuba; 7 Instituto de Biomedicina de Sevilla, Hospital Universitario Virgen del Rocío, Consejo Superior de Investigaciones Científicas/Universidad de Sevilla, 41013 Seville, Spain; 8 Centro de Investigación Biomédica en Red de Epidemiología y Salud Pública, 28029 Madrid, Spain

**Keywords:** bibliometric analysis, *Pneumocystis jirovecii*, research trends

## Abstract

*Pneumocystis jirovecii* is an opportunistic fungal pathogen responsible for *Pneumocystis* pneumonia (PCP), a severe infection that remains a major cause of morbidity and mortality among immunocompromised patients, particularly in non-HIV immunosuppressed populations. Despite its recognized clinical relevance and inclusion in the World Health Organization’s Fungal Priority Pathogens List, important gaps persist in its diagnosis, epidemiology, and therapeutic management. This study provides a comprehensive bibliometric analysis of global scientific production on *P. jirovecii* using Scopus as the primary data source. Publications were evaluated for temporal trends, document types, authorship patterns, institutional productivity, collaboration networks, funding sources, thematic evolution, and journal distribution, with additional comparison against other major pneumonia-associated pathogens. A total of 27,396 articles published between 1916 and 2025 were identified. Over the last 50 years, scientific output increased from 10,382 publications in 1975–2000 to 16,496 in 2001–2025, representing an overall growth of 58.9%. Early research expansion was strongly shaped by the HIV/AIDS epidemic, whereas the post-2000 period reflected advances in molecular diagnostics, taxonomic clarification, and broader attention to non-HIV immunosuppressed populations. Although the field has become more diversified and clinically integrated, persistent structural inequities and underinvestment continue to limit progress, particularly in low- and middle-income settings.

## 1. Introduction

*Pneumocystis jirovecii* (historically referred to as *Pneumocystis carinii* in earlier literature) is an opportunistic fungus with a predominantly pulmonary tropism, capable of causing a potentially life-threatening pneumonia in immunocompromised individuals. *P. jirovecii* was previously considered a parasite and later reclassified as a fungus using molecular methods and phylogenetic analysis [1,2,3].

*Pneumocystis* pneumonia (PCP) typically occurs in patients with impaired cell-mediated immunity, including people living with HIV (PLHIV), patients with malignancies—particularly hematologic neoplasms—transplant recipients, and individuals receiving immunosuppressive therapies, such as prolonged glucocorticoid use in combination with other immunomodulatory agents [4,5].

Among patients with hematologic conditions, those with acute lymphoblastic leukemia, non-Hodgkin lymphoma—especially patients exposed to rituximab-containing regimens—chronic lymphocytic leukemia, multiple myeloma, and recipients of allogeneic hematopoietic stem cell transplantation are particularly prone to PCP [6,7].

From a historical perspective, *Pneumocystis* gained epidemiological relevance following its identification as a cause of pneumonia in postwar Europe and later achieved global prominence during the HIV epidemic of the 1980s, when PCP became established as a defining opportunistic infection of acquired immunodeficiency syndrome (AIDS) [8,9]. The introduction of primary prophylaxis and the widespread implementation of combined antiretroviral therapy (HAART/cART) led to a substantial decline in the incidence of PCP among HIV-infected populations [10,11]. Nevertheless, the disease continues to represent a major clinical challenge, particularly in settings of HIV-unrelated immunosuppression, where presentation is often more abrupt, the clinical course more fulminant, and mortality significantly higher [5,12].

It is currently estimated that *Pneumocystis* infections have an approximate annual global incidence of 505,000 cases, with an associated mortality rate of 42.4%, corresponding to nearly 214,000 deaths per year [13]. In this context, and in recognition of its significant clinical impact as well as the persistent challenges related to timely diagnosis, effective treatment, and prevention, *P. jirovecii* has been included in the World Health Organization’s Fungal Priority Pathogens List, within the medium-priority group [14]. This classification aims to guide research efforts, the development of diagnostic and therapeutic tools, and the implementation of global public health strategies, with the purpose of reducing the disease burden and mortality associated with this pathogen. In doing so, it seeks to contribute to the achievement of Sustainable Development Goal 3, which focuses on ensuring healthy lives and promoting well-being for all populations, particularly through the control of communicable diseases and the reduction in preventable mortality [15].

Beyond its well-established role as the etiological agent of PCP, *P. jirovecii* has also been detected as a respiratory colonizer in several chronic pulmonary diseases, including chronic obstructive pulmonary disease (COPD), with reported prevalence rates ranging from 16% to 55% [16,17]; interstitial lung disease (ILD), with colonization reported in 33.8% of patients [18]; cystic fibrosis (CF), with frequencies ranging from 2.5% to 38.2% [19,20]; asthma, where a pilot study detected *Pneumocystis* DNA in 20% of patients [21]; and lung cancer, with reported detection rates from 4.3% [22] to 17.9% [23]. Although causality remains unclear, these findings suggest that *P. jirovecii* may have clinical relevance beyond classical PCP, particularly as a potential contributor to airway inflammation, pulmonary exacerbations, impaired lung function, and transmission to susceptible hosts [16,18,19,20,21,22,23]. 

Despite its well-recognized clinical and epidemiological relevance, research on *P. jirovecii* continues to be marked by substantial knowledge gaps. These limitations encompass fundamental aspects of the pathogen’s biology, including its life cycle, the mechanisms regulating the transition from asymptomatic colonization to invasive disease, and the true dynamics of transmission across different populations and healthcare settings [17,24].

Consistent with these fundamental uncertainties, the diagnosis of PCP remains a significant challenge. The standardized differentiation between colonization and active pneumonia is particularly complex in patients without HIV infection, in whom lower fungal burdens compromise the performance and reproducibility of currently available biomarkers [25]. In addition, there is a scarcity of robust evidence linking genetic alterations in *P. jirovecii* to therapeutic failure and adverse clinical outcomes, as well as a limited availability of comparable data on molecular epidemiology at the genotype level and on the occurrence of nosocomial outbreaks [26,27]. 

Across these domains, these gaps are profoundly shaped by the inability to reproducibly culture *P. jirovecii* in vitro, which restricts experimental investigation of its biology, precludes the performance of standardized antifungal susceptibility testing, and limits both the development of new therapeutic strategies and the functional validation of molecular findings [24,28]. 

This information gap is particularly relevant in low- and middle-income countries, where the true burden of PCP remains uncertain or underestimated, and limitations in research resources further exacerbate inequalities in evidence generation and in the comprehensive understanding of the pathogen [29,30].

In this context, the objective of the present study is to conduct a bibliometric analysis of the scientific literature on *P. jirovecii* by integrating three complementary approaches: a descriptive assessment of publication patterns and collaboration networks, a historical overview of the evolution of the field, and an interpretative discussion of current gaps and emerging research priorities. Through this approach, the study aims to identify areas of greater and lesser development, scientific collaboration networks, and emerging lines of research, thereby providing an objective framework to help guide future investigations and clinical and public health strategies in this field.

## 2. Materials and Methods

### 2.1. Reporting Guideline

This bibliometric review was organized and reported according to the preliminary BIBLIO guideline for reporting bibliometric reviews of biomedical literature. The completed BIBLIO checklist is provided as Supplementary Material S1 [31].

### 2.2. Data Source and Bibliographic Time Window

This study used Scopus® https://www.scopus.com (accessed on 9 February 2026). as the primary bibliographic database due to its extensive multidisciplinary coverage, which includes more than 28,000 high-impact scientific journals, as well as the availability of standardized metadata fields suitable for bibliometric and scientometric analyses [32,33]. Scopus shows substantial overlap with Web of Science in its indexing of high-impact journals and also covers a considerable proportion of the biomedical literature included in PubMed. However, its broader and more multidisciplinary scope incorporates additional sources not indexed in these databases. Therefore, Scopus represents a robust and appropriate source for conducting comprehensive bibliometric analyses [34].

The search was conducted using the Advanced Search interface of Scopus, and the dataset was retrieved and exported on 9 February 2026. To assess the historical evolution of scientific production, two distinct temporal windows were considered: 1975–2000 and 2001–2025.

### 2.3. Search Strategy

A structured search strategy was designed to identify scientific literature related to *Pneumocystis jirovecii* and *Pneumocystis* pneumonia (PCP). The query was applied to the Title, Abstract, and Author Keywords fields using the Scopus TITLE-ABS-KEY operator. To capture both current and historical terminology associated with *Pneumocystis* research, the search included terms such as *Pneumocystis jirovecii*, *Pneumocystis jiroveci*, *Pneumocystis carinii*, *Pneumocystis pneumonia*, and pneumocystosis. Abbreviations were avoided to reduce ambiguity and minimize the retrieval of unrelated records. Several preliminary search combinations were evaluated, and the final query was selected because it provided the most comprehensive and specific retrieval for the objectives of this bibliometric analysis. The retrieved records were reviewed at the dataset level to verify their consistency with the scope of the study. No additional manual exclusions were applied beyond the predefined Scopus filters and search criteria.

Exact query used in the Scopus Advanced Search (as executed):

[TITLE-ABS-KEY (“Pneumocystis jirovecii”) OR TITLE-ABS-KEY (“Pneumocystis carinii”) OR TITLE-ABS-KEY (Pneumocystosis) OR TITLE-ABS-KEY (“Pneumocystis pneumonia”)]. 

After the initial search, the bibliometric analysis was restricted to two temporal periods, 1975–2000 and 2001–2025, in order to evaluate changes in scientific output over time. No language restrictions were applied, allowing a comprehensive assessment of the global scientific production on this topic.

### 2.4. Bibliometric Data Extraction

For each retrieved record, bibliographic and bibliometric metadata fields available in Scopus were extracted to support performance analysis and scientific mapping. The indicators analyzed included:

Productivity indicators: year of publication, document type, source title, and subject area.

Collaboration indicators: authorship information, institutional affiliations, and countries/regions, used to assess co-authorship patterns.

Thematic indicators: author keywords and indexed keywords (when available), used for co-occurrence analysis and thematic mapping.

Source indicators: leading journals/sources based on publication volume and citation impact, according to citation counts provided by Scopus.

### 2.5. Bibliometric Data Analysis

A standard bibliometric workflow was adopted, consisting of the following steps:

Retrieval of records from Scopus using the predefined query and filters.

Export of the resulting dataset (9 February 2026).

Descriptive analysis of publication and citation patterns.

Network visualization through scientific mapping.

Descriptive analyses included the assessment of annual publication trends, distribution by document type and subject area, and identification of the most productive and/or most cited sources, countries, institutions, and authors, as appropriate.

Scientific mapping and network visualization were performed using VOSviewer to generate bibliometric network maps, including keyword co-occurrence and country-level co-authorship networks. The bibliometric database was obtained from Scopus and is provided as Supplementary Material S2 to improve reproducibility. For the keyword co-occurrence map, co-occurrence was selected as the type of analysis, all keywords as the unit of analysis, and full counting as the counting method. A minimum threshold of 805 keyword co-occurrences/occurrences was applied to reduce network density and retain the most representative terms.

Before cluster interpretation, terminology harmonization was performed to standardize spelling variants, abbreviations, and related terms, while historically relevant terminology, particularly *Pneumocystis carinii*, was retained and interpreted in relation to the taxonomic transition toward *Pneumocystis jirovecii*. The international collaboration network was generated using Scopus country-level co-authorship metadata and full counting. 

When required, minor graphical adjustments were incorporated using Graphic version 3.1 for Mac (Graphic, Picta, Inc., macOS, Palo Alto, CA, USA), including cluster highlights and thematic labels, without modifying the underlying bibliometric networks, link strengths, node positions, cluster assignments, or clustering results generated by VOSviewer (version 1.6.20).

Unless otherwise stated, no additional manual exclusions were applied beyond the predefined Scopus filters (time window and search strategy). If any post-retrieval relevance screening was conducted—for example, removal of false positives due to terminological ambiguity—the number of excluded records and the exclusion criteria would be explicitly reported in this subsection.

### 2.6. Comparative Bibliometric Analysis of Major Pneumonia-Associated Pathogens (2001–2025)

To contextualize the scientific development of *P. jirovecii* research within the broader landscape of pneumonia-associated pathogens, a comparative bibliometric analysis was conducted focusing on major bacterial, viral, and fungal etiological agents of pneumonia. The analysis was restricted to publications indexed in Scopus between 1 January 2001, and 31 December 2025.

Structured search queries were designed using the TITLE-ABS-KEY field to retrieve publications explicitly addressing pneumonia in association with each pathogen. The search strategy for each pathogen is shown in Supplementary Material S3. 

All searches were conducted on the same date to ensure consistency of database coverage. No language restrictions were applied. Document types were not limited in order to capture the overall volume of scientific production across pathogens.

The primary outcome measure was the total number of publications retrieved for each pathogen during the study period. The objective of this comparative approach was not to perform pathogen-specific epidemiological analysis, but rather to evaluate relative research intensity and thematic prioritization within the global scientific agenda on pneumonia.

Given the exceptional surge in publications following the emergence of the COVID-19 pandemic, results related to SARS-CoV-2 were interpreted with caution and considered separately in the discussion to account for post-2020 publication inflation.

## 3. Results and Discussion

### 3.1. Publication Output and Document Types

The bibliographic search strategy retrieved a total of 27,396 documents published between 1916 and 2025, confirming a sustained scientific output spanning more than a century of research on *Pneumocystis* and PCP.

**Figure 1:**
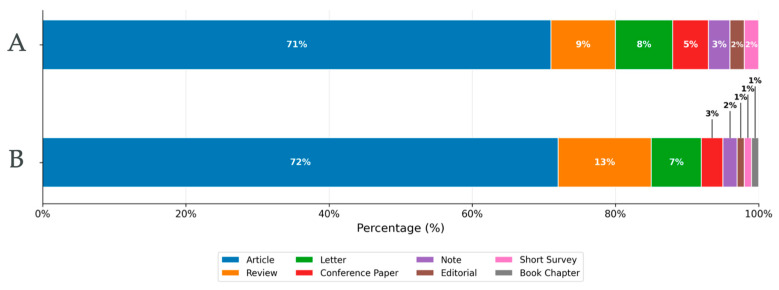
Distribution of document types published on *Pneumocystis* and *Pneumocystis* pneumonia during the periods 1975–2000 (A) and 2001–2025 (B).

When the analysis was restricted to the period 1975–2000, corresponding to the first 25 years of the modern bibliographic window considered in this study, 10,382 publications were identified. Of these, 7377 were original research articles, followed by 951 reviews, 885 letters, 504 conference papers, and 302 notes, while the remaining documents were classified under other document types. During this period, scientific output was predominantly published in English, French, German, and Spanish, reflecting the historical leadership of Europe and North America in this field of research. The distribution of document types across the analyzed time periods is shown in Figure 1. 

In contrast, the analysis of the 2001–2025 period revealed a substantial increase in scientific production, with a total of 16,496 documents published. Within this set, 11,551 were research articles, 2554 were reviews, 970 were letters, 373 were conference papers, and 343 were notes, while the remaining publications were grouped into other categories. During this stage, English became the clearly dominant language, followed by Spanish, and to a lesser extent Chinese, French, and Japanese, suggesting a geographic expansion of scientific interest and increased participation from non-Western regions in contemporary research.

Considering the publications produced between 1975–2000 (n = 10,382) and 2001–2025 (n = 16,496), a substantial increase in scientific output was observed, with an overall growth of approximately 58.9%. This increase was not abrupt but rather sustained over time, as reflected by an average annual growth rate of approximately 1.87% per year, suggesting a progressive and steady expansion.

### 3.2. Publications by Year and the Most Cited Keywords

**Figure 2:**
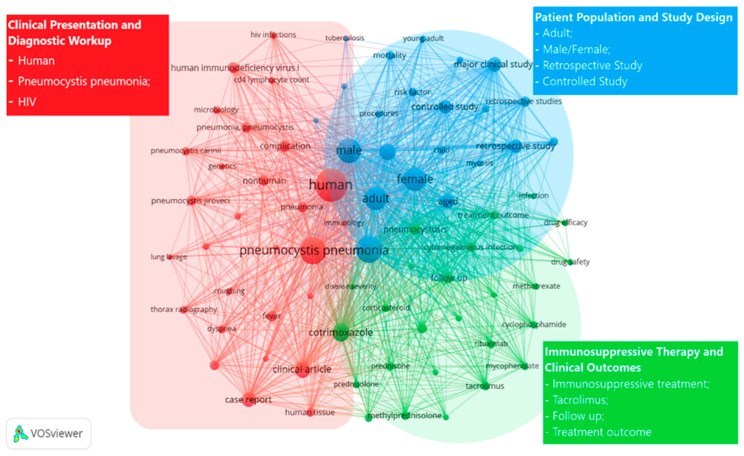
Keyword co-occurrence network and thematic clusters in *Pneumocystis jirovecii* research. Bibliometric network visualization generated with VOSviewer showing the co-occurrence of author and indexed keywords related to *Pneumocystis* pneumonia. Three major thematic clusters are identified: (red) clinical presentation and diagnostic workup, including HIV infection and pneumocystosis; (blue) patient population characteristics and study design, encompassing age, sex, and retrospective or controlled studies; and (green) immunosuppressive therapies and clinical outcomes, highlighting treatment regimens and follow-up. Node size reflects keyword frequency, while link thickness indicates the strength of co-occurrence between terms.

**Figure 3:**
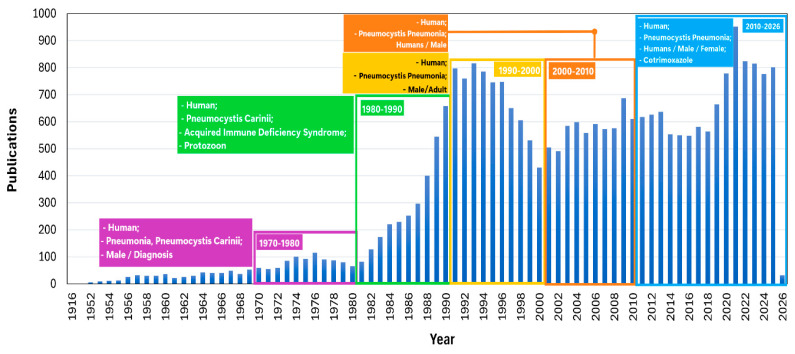
Annual publication trends on *Pneumocystis* and *Pneumocystis* pneumonia (1916–2025), highlighting major shifts in research focus and scientific output across distinct historical periods.

Based on the collected data, a keyword co-occurrence analysis integrated with scientific production over time was performed, allowing the identification of the main thematic clusters and their historical evolution (Figure 2). The decade-based analysis (Figure 3) demonstrates a close correspondence between changes in scientific output and the conceptual development of *Pneumocystis* research between 1916 and 2025.

Between 1975 and 1980, scientific output remained relatively low and stable, reflecting an early exploratory phase dominated by descriptive clinical reports, histopathological observations, and case-based studies. During this period, prevailing themes focused on the basic clinical characterization of PCP in immunocompromised patients, in the absence of a clearly defined global epidemiological framework.

In contrast, the decade from 1981 to 1990 was characterized by a progressive increase in publications, coinciding with the emergence of the HIV/AIDS epidemic. This expansion was accompanied by a broadening of thematic scope toward core biomedical disciplines, including immunology, infectious diseases, and public health. As a result, PCP became firmly established as a key opportunistic infection—a shift that explains both the sustained growth in scientific output and the increasing diversification of research approaches during this period.

The temporal analysis of scientific output on *Pneumocystis* and pneumocystosis shows sustained growth throughout the twentieth century, interrupted by a transient plateau toward the late 1990s. 

In particular, between 1997 and 2000, a relative decline in the number of publications was observed, giving rise to a non-linear dynamic characterized by a brief stagnation followed by a sustained rebound in subsequent decades (Figure 3). This pattern should not be interpreted as direct evidence of a single causal process. However, it is temporally consistent with major transformations in the clinical and epidemiological landscape of PCP, particularly the introduction and rapid expansion of highly active antiretroviral therapy (HAART) in high-income countries and the widespread use of primary prophylaxis with cotrimoxazole. These interventions were associated with substantial reductions in PCP incidence and mortality among people living with HIV [10,35,36,37,38], and may have influenced research priorities related to HIV-associated PCP during this period. Nevertheless, bibliometric curves cannot by themselves prove this relationship, and the observed plateau should therefore be interpreted cautiously as a pattern potentially shaped by multiple clinical, methodological, and research-system factors.

In this context, the disease ceased to be perceived as a dominant clinical emergency and came to be regarded as a largely controlled opportunistic infection, generating a perception of a “resolved problem” that reduced clinical urgency and the incentive for new therapeutic and epidemiological studies, thereby contributing to a transient contraction in the volume of publication [10,38,39]. This effect was further reinforced by a transitional technological gap during the same period, as molecular biology tools were not yet sufficiently standardized or widely available. This limitation constrained the formulation of new research questions related to fungal burden quantification, the differentiation between colonization and active infection, and the exploration of the pathogen’s genetic diversity [40,41]. 

In contrast, the sustained increase in publications from the early 2000s onward reflects a qualitative reconfiguration of the field. The progressive standardization of molecular techniques—particularly PCR and later real-time PCR—redefined diagnostic approaches and enabled new lines of research focused on pulmonary colonization, fungal burden dynamics, and pathogen detection in less invasive samples, thereby substantially expanding the range of clinical and epidemiological questions that could be investigated [42,43]. In parallel, the taxonomic recognition of *P. jirovecii* as the species specific to humans prompted conceptual revisions, reinterpretations of historical series, and comparative studies, reinforcing its identity as a highly specialized, strictly host-dependent fungus [1,44,45,46].

This was accompanied by a shift in the epidemiological profile of the disease. In the post-HAART era, pneumocystosis gained greater relative relevance in non-HIV populations—including transplant recipients, patients with malignancies, those receiving immunosuppressive therapies, or critically ill individuals—in whom clinical presentation is often more severe and diagnosis more delayed, reintroducing *Pneumocystis* as an ongoing clinical challenge and stimulating observational, prognostic, and therapeutic management studies [11,12,47,48]. Moreover, it became evident that the benefits of antiretroviral therapy critically depended on treatment adherence and the appropriate management of therapeutic regimens [35]. Exposure to prior monotherapies, treatment interruptions, and suboptimal adherence favored the selection of resistant viral variants, leading to virological failure and incomplete recovery of cellular immunity [49]. In this context, these factors may also have contributed, to a lesser extent, to the occurrence of opportunistic infections, including PCP, thereby helping to reactivate clinical and epidemiological interest in the disease [10].

Finally, between 2021 and 2025, scientific production remained at high levels, with moderate year-to-year fluctuations. Figure 3 suggests a stabilization of the field at a high productivity threshold, with a thematic emphasis oriented toward clinical outcomes, therapeutic management, and emerging research contexts. During this period, a transient increase in publications associated with the COVID-19 pandemic is also observed [50,51]. The apparent decline observed in the final year should be interpreted with caution, as it is likely attributable to delays in indexing processes rather than to a genuine reduction in scientific activity.

### 3.3. Authors

The analysis of the most prolific authors by temporal periods (Table 1) reveals a clear differentiation between the foundational phase of the field (1975–2000) and its stage of consolidation and diversification (2001–2025). In this context, productivity refers to the volume of scientific output, measured by the number of publications, whereas impact reflects citation-based influence, assessed through total citations, mean citations per publication, and h-index. This distinction helps differentiate prolific authors from those whose work achieved greater bibliometric visibility within the field. During the 1975–2000 period, scientific production was markedly concentrated among a small group of pioneering researchers, led by Walter T. Hughes, Peter D. Walzer, and Henry Masur. These authors combined high productivity with substantial scientific impact, as reflected by elevated average citation counts and high h-index values. This core group was predominantly affiliated with academic and clinical institutions in the United States, underscoring the central role of this country in the early generation of knowledge on *Pneumocystis*.

In contrast, the 2001–2025 period shows a partial reconfiguration of scientific leadership, led by Andrew Harold Limper and David W. Denning, alongside the continued presence of previously established authors and the emergence of new key contributors. This stage is characterized by greater geographic diversity, with a more visible presence of European institutions—particularly from the United Kingdom and France—as well as a sustained increase in average author impact, suggesting enhanced international visibility and influence of research during this period. 

Taken together, these patterns reflect not only the historical evolution and thematic maturation of the field, but also a persistent geographic asymmetry in knowledge production. The concentration of scientific leadership in high-income countries highlights the need to promote and strengthen the development of leaders in the field of mycoses in low- and middle-income countries, where these infections remain closely associated with underdiagnosis, limited diagnostic infrastructure and funding, and consequently high morbidity and mortality rates. From this perspective, identifying these gaps represents a strategic opportunity to guide capacity-building efforts, international collaboration, and the generation of locally relevant evidence in settings with a higher disease burden.

### 3.4. Most Productive Countries

**Figure 4:**
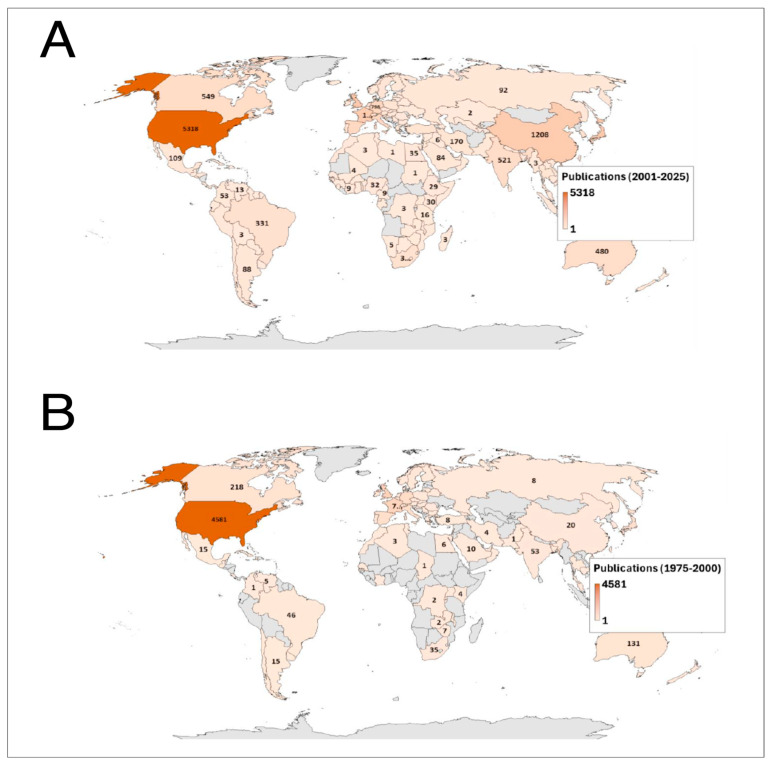
Global distribution of *Pneumocystis* research output by country during 1975–2000 (**B**) and 2001–2025 (**A**).

The geographic distribution of scientific production on *Pneumocystis* shows a marked concentration in a limited number of countries across both study periods (Figure 4). Between 1975 and 2000, the United States dominated global output (n = 4581), followed at a considerable distance by Canada (n = 218) and Australia (n = 131). European countries such as France, Germany, the United Kingdom, Italy, and Spain showed moderate but comparatively lower contributions, while production from Latin America, Africa, and most of Asia remained limited during this initial phase.

In contrast, the 2001–2025 period reveals both consolidation and geographic diversification of research activity. Although the United States maintained its leading position (n = 5318), substantial growth was observed in China (n = 1208), Canada (n = 549), India (n = 521), Australia (n = 480), and Brazil (n = 331). European countries collectively increased their output, while a noticeable expansion of scientific contributions emerged from Asian countries, the Middle East, and South America. This increase in publication output, particularly during the most recent years (2013–2025 period), may reflect not only a growing scientific interest in *Pneumocystis* research, but also broader trends in global biomedical publishing, including the expansion of indexed journals and the increasing contribution of highly productive research groups from Asia and other emerging scientific regions. Despite this broader participation, research productivity remains unevenly distributed, with North America, Western Europe, and selected Asian countries accounting for most global publications.

**Figure 5:**
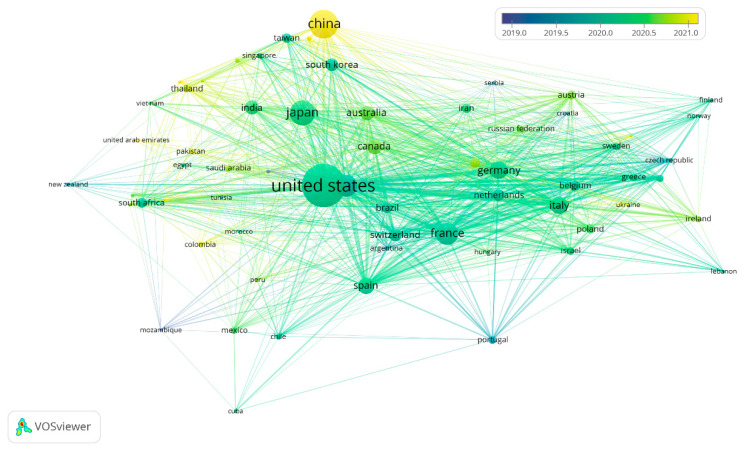
International collaboration network in *Pneumocystis* research during the most recent study period. Node size is proportional to the volume of publications by country, link thickness reflects the strength of collaborative ties, and node color indicates the average year of publication.

When evaluating the international collaboration network analysis for the most recent years, a highly centralized structure is observed, with the United States functioning as the main global hub (Figure 5). European countries form a dense and highly cohesive collaborative cluster, characterized by strong intra-regional interactions. In parallel, Asian countries—particularly China, Japan, and South Korea—exhibit rapid growth and increasing centrality within the network, reflecting their progressive consolidation in the field. In contrast, Latin America, Africa, and the Middle East remain in more peripheral positions, with collaboration patterns largely mediated through partners in North America and Europe, highlighting the persistence of structural asymmetries in global scientific production (Figure 5).

### 3.5. Most Productive Institutions 

**Figure 6:**
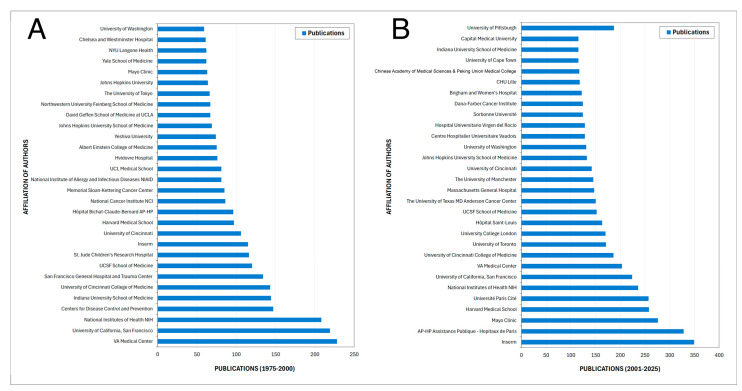
Leading institutions in *Pneumocystis* research during 1975–2000 (**A**) and 2001–2025 (**B**).

The analysis of the most productive institutions across the 1975–2000 and 2001–2025 periods reveal a profound reorganization of institutional leadership in *Pneumocystis* research (Figure 6A,B). During the earlier period, scientific output was predominantly concentrated in United States–based institutions, particularly federal agencies and major academic medical centers such as the National Institutes of Health (NIH), VA Medical Centers, the Centers for Disease Control and Prevention (CDC), and universities with strong clinical traditions. This pattern reflects an initial phase of the field closely linked to the epidemiological and clinical response to the HIV/AIDS epidemic, in which knowledge generation was largely driven by public health priorities and descriptive clinical research.

In contrast, the 2001–2025 period shows a shift in the axis of productivity toward hospital-based networks and national research platforms, with a particularly strong emergence of European institutions. Notably, Inserm and the Assistance Publique–Hôpitaux de Paris lead in publication output and clearly exceed the maximum productivity levels observed in the earlier period (Figure 6). This shift suggests a structural transition of the field toward a phase of clinical and molecular consolidation, supported by large hospital cohorts, transplant and oncology programs, and other forms of non–HIV-related immunosuppression.

Despite this reconfiguration, a stable institutional core—including Mayo Clinic, Harvard Medical School, UCSF, the NIH, and the University of Cincinnati—remains prominent across both periods. This continuity indicates that sustained productivity in the field depends on advanced diagnostic infrastructure, access to complex clinical populations, and long-term continuity in research programs. At the same time, the reduced relative prominence of public health agencies in the later period reinforces the notion of a progressive shift toward high-complexity hospital settings.

Finally, Figure 6 also highlights a geographical expansion of the field, with the inclusion of leading institutions from Asia and Africa in the second period. Taken together, these findings indicate that the evolution of *Pneumocystis* research has been not only quantitative but also structural, transitioning from a concentrated and reactive model to a multinodal, internationalized, and clinically integrated research landscape.

### 3.6. Journals 

The comparison of the most prolific journals between 1975–2000 and 2001–2025 highlights a substantial transformation in the publication landscape of *Pneumocystis* research (Table 2). In this analysis, journal productivity was interpreted as the volume of *Pneumocystis*-related publications, whereas journal impact was assessed through citation-based indicators, including total citations and mean citations per publication. Journal quartile, according to Scimago, was used as a complementary indicator of journal-level visibility and editorial positioning, rather than as a direct measure of the impact of individual articles.

During the earlier period, scientific output was dominated by high-impact general medical and infectious disease journals, including *Chest*, *The New England Journal of Medicine*, The *Lancet*, *Annals of Internal Medicine*, and *Journal of Infectious Diseases*, reflecting the central clinical relevance of PCP during the HIV/AIDS epidemic.

In parallel, the presence of specialized journals such as *Journal of Protozoology* and *Journal of Eukaryotic Microbiology* points to an early taxonomic and biological focus, when the classification and biology of the organism were still under active investigation, positioning the field at the interface between clinical medicine and fundamental biology.

By contrast, the 2001–2025 period shows a clear diversification and specialization of publication venues (Table 2). While *Clinical Infectious Diseases* remains a core outlet, there is a marked rise in transplantation- and hematology-focused journals, including *Transplant Infectious Disease*, *Bone Marrow Transplantation*, *Biology of Blood and Marrow Transplantation*, and *Leukemia and Lymphoma*, consistent with the epidemiological shift in *Pneumocystis* toward non-HIV immunosuppressed populations.

Additionally, the growing presence of case-report–oriented and open-access journals, such as *BMJ Case Reports* and *BMC Infectious Diseases*, suggests a fragmentation of the evidence base, with greater emphasis on heterogeneous clinical scenarios and rare presentations. The persistence of *Journal of Eukaryotic Microbiology*, albeit with reduced citation impact, indicates that fundamental biological research continues but now plays a complementary role within a predominantly clinical framework.

Overall, these patterns reflect the maturation of the field, characterized by a shift from generalist medical journals toward specialized clinical niches and a redefinition of how and where scientific impact in *Pneumocystis* research is generated and disseminated.

### 3.7. Sponsor 

**Figure 7:**
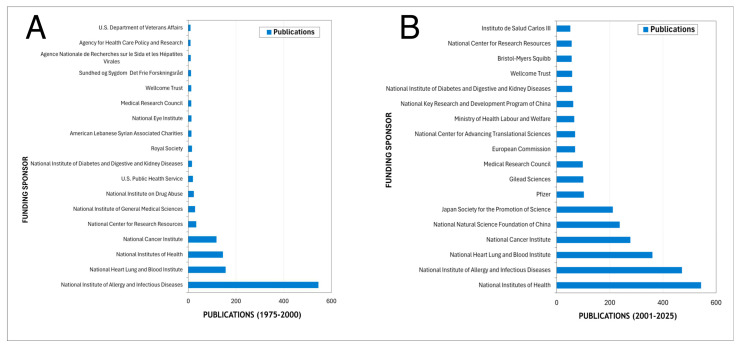
Leading funding agencies supporting *Pneumocystis* research across two study periods. Bar charts showing the most frequent funding sponsors acknowledged in publications indexed in Scopus during 1975–2000 (**A**) and 2000–2025 (**B**).

The analysis of the main funding sponsors reveals a strong predominance of United States federal agencies across both study periods (Figure 7). Between 1975 and 2000, funding was markedly concentrated in the National Institute of Allergy and Infectious Diseases (NIAID), which overwhelmingly led the number of sponsored publications, followed by the National Heart, Lung, and Blood Institute (NHLBI), the National Institutes of Health (NIH), and the National Cancer Institute (NCI). Other contributors included additional NIH-affiliated institutes and a limited number of non-U.S. organizations, indicating a highly centralized funding structure during the foundational phase of the field.

In contrast, the 2000–2025 period shows both consolidation and diversification of funding sources. While the NIH and NIAID remain the leading sponsors, a broader range of international agencies emerges, including the National Natural Science Foundation of China, the Japan Society for the Promotion of Science, the European Commission, and Instituto de Salud Carlos III. Additionally, industry sponsors such as Pfizer, Gilead Sciences, and Bristol-Myers Squibb appear among the most active contributors. This shift reflects a progressive internationalization of financial support and increased participation of Asian and European funding bodies, alongside sustained U.S. leadership.

### 3.8. Bibliometric Positioning of Pneumocystis jirovecii Relative to Other Pneumonia-Associated Pathogens

**Figure 8:**
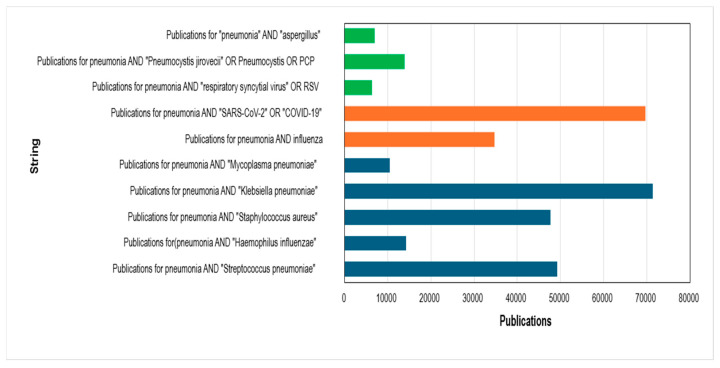
Comparative bibliometric output of major pneumonia-associated pathogens (2000–2025). Bar chart illustrating the number of publications indexed in Scopus that address pneumonia in association with major bacterial, viral, and fungal etiological agents during the study period. Colors indicate pathogen groups: green for fungal pathogens, orange for viruses, and blue for bacteria.

The comparative bibliometric analysis of pneumonia-associated pathogens reveals a marked imbalance in scientific production across etiological agents (Figure 8). As shown in Figure 8, bacterial pathogens such as *Klebsiella pneumoniae*, *Streptococcus pneumoniae*, and *Staphylococcus aureus* account for the largest share of publications, followed by viral agents, particularly SARS-CoV-2, which exhibits an unprecedented surge in output within a remarkably short time frame. In contrast, *P. jirovecii* remains substantially underrepresented in the global research landscape.

**Figure 9:**
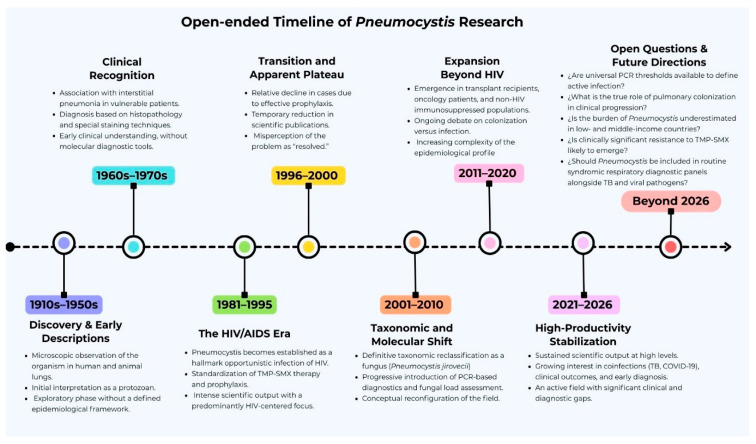
Timeline summarizing the historical evolution of knowledge on *Pneumocystis jirovecii*.

Notably, despite its relatively recent emergence, SARS-CoV-2 rapidly accumulated a volume of publications comparable to—or even exceeding—that of long-established bacterial causes of pneumonia (Figure 9). This phenomenon reflects the rapid mobilization of global funding, coordinated international research efforts, and prioritization at the highest public health levels during the COVID-19 pandemic. By comparison, pneumocystosis—although recognized for decades as a major opportunistic infection and associated with significant mortality in immunocompromised populations—has not experienced a proportional expansion in research output, as evidenced by the comparatively lower publication counts observed in Figure 8.

The disparity illustrated in Figure 9 likely reflects structural asymmetries in research investment rather than differences in clinical relevance alone. While the global response to COVID-19 demonstrated how rapidly scientific production can expand when substantial financial and political support is mobilized, pneumocystosis continues to receive comparatively limited attention despite its persistent burden among vulnerable populations. Given the growing number of immunosuppressed individuals worldwide—including transplant recipients, oncology patients, and individuals receiving biologic or immunomodulatory therapies—this imbalance raises concerns regarding whether current funding priorities adequately reflect evolving epidemiological realities. Strengthening investment in pneumocystosis research is therefore essential to ensure advances in diagnostics, prophylaxis, and therapeutic strategies are aligned with its ongoing clinical impact.

## 4. Final Considerations

The evolution of *Pneumocystis* research reflects a dynamic and increasingly complex field, shaped by changing epidemiological patterns and major advances in diagnostic technologies. As illustrated in Figure 9, knowledge has progressed from early microscopic observations and the initial clinical recognition of *Pneumocystis* pneumonia (PCP), through the pivotal HIV/AIDS era, to the taxonomic reclassification of *P. jirovecii* as a fungus and the consolidation of molecular diagnostic approaches. More recently, the growing burden of PCP among non-HIV immunosuppressed populations has redefined the epidemiological profile of this infection and highlighted new clinical, diagnostic, and therapeutic challenges.

Despite the sustained expansion of scientific output, important structural and conceptual gaps continue to limit progress in the field. Among these, the lack of robust and reproducible in vitro culture systems remains one of the main technical barriers, restricting a deeper understanding of the biology of *P. jirovecii*, its interaction with the host immune system, drug susceptibility testing, and the identification of new diagnostic and therapeutic targets [24]. In parallel, distinguishing colonization from active infection continues to be a major unresolved issue, particularly in the era of highly sensitive molecular assays [52]. This challenge is especially relevant in non-HIV patients, in whom the fungal burden is often low and the clinical presentation may differ substantially from that of classical HIV-associated PCP [6].

In addition, the clinical significance of *Pneumocystis* colonization in non-HIV patients with respiratory diseases other than pneumonia remains incompletely understood. Recent studies have suggested a possible association between *Pneumocystis* colonization and chronic respiratory diseases, such as asthma and chronic obstructive pulmonary disease. Likewise, some authors have proposed that this fungus may act as a cofactor in symptom exacerbation or disease progression [16,53,54]. However, this relationship remains hypothetical, and further studies are needed to clarify the role of *Pneumocystis* in these conditions.

At the diagnostic level, the field has evolved from conventional microscopy toward more sensitive molecular methods, with quantitative real-time PCR currently being one of the most widely used tools for detection [55,56]. However, further validation of less invasive specimens, including induced sputum, nasopharyngeal aspirates, and oral wash samples, as well as emerging approaches such as droplet digital PCR, CRISPR-based assays, and blood biomarkers such as serum β-D-glucan, will be essential to improve early and accurate diagnosis [55,57,58,59,60]. Therapeutically, trimethoprim/sulfamethoxazole remains the treatment of choice, but its frequent dose-dependent adverse events and the absence of routine culture-based susceptibility testing limit direct resistance assessment, underscoring the need for safer regimens and improved prophylactic and treatment strategies [61,62].

Although stewardship interventions specifically directed at PCP management are not yet widely standardized, current principles for optimizing antifungal and antimicrobial use can be applied through diagnostic stewardship, optimization of prophylaxis and treatment, and audits of adherence to clinical guidelines [63,64]. Tools such as the EQUAL *Pneumocystis* Score and diagnostic algorithms integrating PCR for *P. jirovecii*, chest imaging, and serum β-D-glucan may support more accurate clinical decision-making, rational drug use, and cost containment in high-risk populations [60,65,66]. In addition, guidelines adapted to different clinical presentations and risk populations could improve patient management, optimize the use of trimethoprim/sulfamethoxazole as first-line therapy, and reduce unnecessary or prolonged antimicrobial exposure, thereby contributing to preserving its therapeutic efficacy [65,67,68].

Beyond diagnosis, treatment, and stewardship, several broader research priorities remain. A better understanding of transmission dynamics, including the role of asymptomatic carriers, nosocomial spread, and possible vertical transmission, is still needed to clarify the true epidemiological impact of *P. jirovecii* [69]. At the same time, the incorporation of artificial intelligence into radiological and clinical decision-making, along with the expansion of multi-omics approaches, offers promising opportunities to refine diagnosis, uncover mechanisms of pathogenesis, and identify new intervention targets [70]. These integrative strategies may be particularly valuable for understanding how *P. jirovecii* interacts with the pulmonary microbiome, host immunity, and chronic respiratory disease processes [71].

Overall, future progress in *Pneumocystis* research will depend on multidisciplinary efforts that combine epidemiology, molecular diagnostics, cell biology, clinical research, and computational approaches. Advancing knowledge in these areas will not only improve our understanding of this highly adapted opportunistic pathogen, but also support the development of more effective strategies for the prevention, diagnosis, and management of PCP. Ultimately, accurate interpretation of *P. jirovecii* detection will continue to require careful integration of clinical findings with highly sensitive laboratory methods.

## 5. Conclusions

In conclusion, this bibliometric analysis shows that research on *Pneumocystis jirovecii* has expanded substantially over time, evolving from an early phase dominated by the HIV/AIDS epidemic to a more diversified and clinically integrated field increasingly focused on non-HIV immunosuppressed populations. Nevertheless, within the limits of bibliometric interpretation, this growth appears not to have been matched by proportional research prioritization when compared with other major pneumonia-associated pathogens, and important structural inequities in scientific leadership, funding, and geographic participation remain apparent.

Despite important advances in molecular diagnostics and a broader understanding of the epidemiological profile of PCP, major unresolved challenges continue to constrain progress, particularly the absence of reproducible *in vitro* culture systems, the difficulty in distinguishing colonization from active disease, and the need for more accurate, less invasive, and clinically interpretable diagnostic strategies. These gaps are especially relevant in low- and middle-income settings, where underdiagnosis and limited research capacity may contribute to masking the true burden of disease.

Taken together, our findings suggest that the future of *P. jirovecii* research will depend on stronger multidisciplinary collaboration, more equitable global investment, and the integration of molecular, clinical, epidemiological, and computational approaches. Strengthening these areas will be important to advance pathogen biology, improve diagnostic precision, refine prevention and treatment strategies, and help ensure that scientific priorities are better aligned with the persistent clinical impact of pneumocystosis worldwide.

## 6. Strengths and Limitations of the Study

This study has several strengths. First, it provides a comprehensive bibliometric overview of *P. jirovecii* research over a broad historical period, allowing the identification of long-term trends in scientific production, thematic evolution, geographic distribution, institutional leadership, funding patterns, and international collaboration. Second, the use of Scopus as the primary data source allowed the retrieval of standardized bibliographic metadata suitable for bibliometric and scientometric analyses. Third, the study incorporated multiple complementary indicators, including publication output, document types, authors, countries, institutions, journals, sponsors, keyword co-occurrence, and collaboration networks, providing a multidimensional view of the field. Finally, the comparative analysis with other major pneumonia-associated pathogens helped contextualize the relative position of *P. jirovecii* within the broader landscape of pneumonia research.

Nevertheless, this study also has limitations inherent to the bibliometric approach employed. The analysis was based exclusively on the Scopus database; therefore, scientific output indexed only in other platforms was not considered. The lack of comparison with additional sources such as Web of Science, Google Scholar, PubMed, Scientific Electronic Library Online (SciELO), Cochrane Library, or Embase may limit the comprehensiveness of the bibliographic landscape evaluated. Furthermore, the selection of search terms and the strategy applied within Scopus may have introduced retrieval bias. Consequently, the findings should be interpreted as a structured but partial representation of the existing body of literature on the topic. 

## Figures and Tables

**Table 1:** Most prolific authors in *Pneumocystis* research according to Scopus during the periods 1975–2000 and 2001–2025.

Top 10 Most Prolific Authors in Scopus (1975–2000)							Top 10 Most Prolific Authors in Scopus (2001–2025)						
Rank	Author/Scopus ID	Affiliation	Country	n Pub	Citations/Mean Citations	Hi	Rank	Author	Affiliation/Scopus ID	Country	n Pub	Citations/Mean Citations	Hi
**1**	Walter T. Hughes 7202094239	St. Jude Children’s Research Hospital	United States	115	4911/42.7	34	**1**	Andrew Harold Limper 7006099760	Mayo Clinic	United States	112	5153/46	31
**2**	Peter D. Walzer 7007081824	University of Cincinnati	United States	101	3697/36.6	37	**2**	David W. Denning 7102640098	The University of Manchester	United Kingdom	109	10,534	33
**3**	Henry Masur 56744299400	NIH Clinical Center (CC)	United States	100	7440/74.4	42	**3**	Melanie T. Cushion 35474601800	University of Cincinnati College of Medicine	United States	97	2297	29
**4**	Marilyn S. Bartlett 7102151507	Indiana University School of Medicine	United States	94	2766/29.4	29	**4**	Laurence Huang 56144978300	UCSF School of Medicine	United States	90	4327	33
**5**	Queener, Sherry F. 7006547726	Indiana University School of Medicine	United States	91	3382/37.1	35	**5**	Gilles Nevez 7003589276	Université de Bretagne Occidentale	France	73	1369/18.7	21
**6**	James W. Smith 9844027900	Indiana University School of Medicine	United States	90	2728/30.3	30	**6**	Joseph A. Kovacs 7202246288	National Institutes of Health Clinical Center	United States	71	2684/37.8	28
**7**	Robert F Miller 55547118657	University College London	United Kingdom	69	2000/29	25	**7**	Eduardo Dei-Cas 7006604602	Center for Infection and Immunity of Lille (CIIL)	France	60	1981/33	26
**8**	Eduardo Dei-Cas 7006604602	Center for Infection and Immunity of Lille (CIIL)The institution will open in a new tab, Lille, France	France	65	1774/27	27	**8**	Chaohung Lee 7410145264	Indiana University School of Medicine	United States	58	1141/19.6	20
**9**	Melanie T. Cushion 35474601800	University of Cincinnati College of Medicine	United States	62	1959/31.5	28	**9**	Theodore J. Kottom 6603350625	Mayo Clinic, Rochester	United States	55	1079/19.6	17
**10**	Joseph A. Kovacs 7202246288	National Institutes of Health Clinical Center	United States	60	5097/84.9	34	**10**	Robert F. Miller 55547118657	University College London	United Kingdom	55	2331/42.3	24

**Table 2:** Top 10 most prolific journals publishing *Pneumocystis* research indexed in Scopus during the periods 1975–2000 and 2001–2025.

Top 10 Most Prolific Journals in Scopus (1975–2000)						Top 10 Most Prolific Journals in Scopus (2001–2025)			
Ranking	Journal	Pub. n	Total Citations/Mean Citations (1996–2000) *	Quartile (*Scimago*)	Ranking	Journal	Pub. n	Total Citations/Mean Citations (2001–2025)	Quartile (*Scimago*)
**1**	Chest	326	9032/27.7	Q1	1	Clinical Infectious Diseases	239	16,762/77.6	Q1
**2**	Clinical Infectious Diseases	228	9290/40	Q1	2	Transplant Infectious Disease	228	4325/18.9	Q2
**3**	New England Journal of Medicine	216	40,602/187	Q1	3	Bone Marrow Transplantation	210	7383/35.1	Q1
**4**	Journal of Eukaryotic Microbiology	202	2771/13.7	Q2	4	AIDS	165	6491/42.7	Q1
**5**	AIDS	193	7289/37.7	Q1	5	BMJ Case Reports	159	673/4.2	Q4
**6**	Lancet	191	7497/39.2	Q1	6	Transplantation Proceedings	153	1817/11.8	Q3
**7**	Annals of Internal Medicine	180	14,542/80.7	Q1	7	Biology of Blood and Marrow Transplantation	152	8916/58.6	Q1
**8**	Journal of Infectious Diseases	159	8487/53.3	Q1	8	Journal of Eukaryotic Microbiology	137	1302/9.5	Q1
**9**	Antimicrobial Agents and Chemotherapy	134	5264/39	Q1	9	BMC Infectious Diseases	130	2144/16.4	Q1
**10**	Journal of Protozoology	125	1562/12.7	Q1	10	Leukemia and Lymphoma	121	2316/19.1	Q2

* Scopus citation information is available only from 1996 onward. Accordingly, while the document search covered the period 1975–2000, total and mean citation values could only be calculated for publications from 1996 to 200.

## Data Availability

The original contributions presented in this study are included in the article/Supplementary Materials. Further inquiries can be directed to the corresponding authors.

## Supplementary Materials

The following supporting information can be downloaded at: https://www.mdpi.com/article/10.3390/pathogens15050530/s1, Supplementary Material S1: Completed BIBLIO checklist. This supplementary material presents the completed BIBLIO checklist used to guide the organization and reporting of this bibliometric review, documenting compliance with the recommended items for biomedical bibliometric reviews. Supplementary Material S2. Bibliometric database and VOSviewer parameters used for scientific mapping and network visualization. This supplementary file contains the bibliometric database retrieved from Scopus and used for the scientific mapping and network visualization analyses performed in VOS viewer. The dataset was used to generate bibliometric network maps, including keyword co-occurrence and country-level co-authorship networks. For the keyword co-occurrence analysis, co-occurrence was selected as the type of analysis, all keywords as the unit of analysis, and full counting as the counting method. A minimum threshold of 805 keyword co-occurrences/occurrences was applied to reduce network density and retain the most representative terms, thereby improving the interpretability and reproducibility of the bibliometric analysis. Supplementary Material S3: Search strategies used for the comparative bibliometric analysis of pneumonia-associated pathogens. This supplementary material S3 presents the Scopus search strategies applied to identify publications related to major pneumonia-associated pathogens included in the comparative bibliometric analysis. For each pathogen, a specific TITLE-ABS-KEY query was designed by combining the term *pneumonia* with the corresponding pathogen name or commonly used alternative terms. The pathogens assessed were *Streptococcus pneumoniae*, *Haemophilus influenzae*, *Staphylococcus aureus*, *Klebsiella pneumoniae*, *Mycoplasma pneumoniae*, influenza virus, SARS-CoV-2/COVID-19, respiratory syncytial virus (RSV), *Pneumocystis jirovecii*/Pneumocystis pneumonia (PCP), and *Aspergillus* spp. These search strategies were used to ensure a standardized and reproducible comparison of scientific output across relevant infectious agents associated with pneumonia.

## References

[1] Stringer J.R., Beard C.B., Miller R.F., Cushion M.T. (2003). A new name (*Pneumocystis jiroveci*) for Pneumocystis from human. Emerg. Infect. Dis..

[2] Wilkin A., Feinberg J. (1999). Pneumocystis carinii pneumonia: A clinical review. Am. Fam. Physician.

[3] Catherinot E., Lanternier F., Bougnoux M.-E., Lecuit M., Couderc L.-J., Lortholary O. (2010). *Pneumocystis jirovecii* pneumonia. Infect. Dis. Clin..

[4] Kim S.J., Lee J., Cho Y.-J., Park Y.S., Lee C.-H., Yoon H.I., Lee S.-M., Yim J.-J., Lee J.H., Yoo C.-G. (2014). Prognostic factors of *Pneumocystis jirovecii* pneumonia in patients without HIV infection. J. Infect..

[5] Salzer H.J., Schäfer G., Hoenigl M., Günther G., Hoffmann C., Kalsdorf B., Alanio A., Lange C. (2018). Clinical, diagnostic, and treatment disparities between HIV-infected and non-HIV-infected immunocompromised patients with *Pneumocystis jirovecii* pneumonia. Respiration.

[6] Lee R., Huh K., Kang C.K., Kim Y.C., Kim J.H., Kim H., Park J.S., Park J.Y., Sung H., Jung J. (2025). Diagnosis of *Pneumocystis jirovecii* pneumonia in non-HIV immunocompromised patient in Korea: A review and algorithm proposed by expert consensus group. Infect. Chemother..

[7] Pulsipher A.M., Vikram H.R., Gotway M.B., Cartin-Ceba R., Henry K.J., Johnson A.M., Gassner R.R., Zhou K., Green D.B., Thompson E.R. (2025). An observational analysis of a large cohort of *Pneumocystis jirovecii* pneumonia. Open Forum Infect. Dis..

[8] Stringer J.R. (1996). Pneumocystis carinii: What is it, exactly?. Clin. Microbiol. Rev..

[9] Masur H., Michelis M.A., Greene J.B., Onorato I., Vande Stouwe R.A., Holzman R.S., Wormser G., Brettman L., Lange M., Murray H. (1981). An outbreak of community-acquired Pneumocystis carinii pneumonia: Initial manifestation of cellular immune dysfunction. N. Engl. J. Med..

[10] Morris A., Lundgren J.D., Masur H., Walzer P.D., Hanson D.L., Frederick T., Huang L., Beard C.B., Kaplan J.E. (2004). Current epidemiology of Pneumocystis pneumonia. Emerg. Infect. Dis..

[11] Xue T., Kong X., Ma L. (2023). Trends in the epidemiology of Pneumocystis pneumonia in immunocompromised patients without HIV infection. J. Fungi.

[12] Ibrahim A., Chattaraj A., Iqbal Q., Anjum A., Rehman M., Aijaz Z., Nasir F., Ansar S., Zangeneh T.T., Iftikhar A. (2023). *Pneumocystis jiroveci* pneumonia: A review of management in human immunodeficiency virus (HIV) and non-HIV immunocompromised patients. Avicenna J. Med..

[13] Ahmed S.A., Ismail M., Albirair M., Nail A.M.A., Denning D.W. (2023). Fungal infections in Sudan: An underestimated health problem. PLoS Negl. Trop. Dis..

[14] World Health Organization (2022). WHO Fungal Priority Pathogens List to Guide Research, Development and Public Health Action.

[15] United Nations Transforming Our World: The 2030 Agenda for Sustainable Development. https://sdgs.un.org/2030agenda.

[16] Morris A., Norris K.A. (2012). Colonization by *Pneumocystis jirovecii* and its role in disease. Clin. Microbiol. Rev..

[17] Vera C., Rueda Z.V. (2021). Transmission and colonization of *Pneumocystis jirovecii*. J. Fungi.

[18] Vidal S., De la Horra C., Martín J., Montes-Cano M., Rodríguez E., Respaldiza N., Rodríguez F., Varela J.M., Medrano F.J., Calderón E.J. (2006). *Pneumocystis jirovecii* colonisation in patients with interstitial lung disease. Clin. Microbiol. Infect..

[19] Morilla R., Medrano F.J., Calzada A., Quintana E., Campano E., Friaza V., Calderón E.J., de la Horra C. (2021). *Pneumocystis jirovecii* among patients with cystic fibrosis and their household members. Med. Mycol..

[20] Bonnet P., Le Gal S., Calderon E., Delhaes L., Quinio D., Robert-Gangneux F., Ramel S., Nevez G. (2020). *Pneumocystis jirovecii* in Patients With Cystic Fibrosis: A Review. Front. Cell. Infect. Microbiol..

[21] Davey E.L., Colombo R.E., Fiorentino C., Fahle G., Davey R.T., Olivier K.N., Kovacs J.A. (2017). Pneumocystis colonization in asthmatic patients not receiving oral corticosteroid therapy. J. Investig. Med..

[22] Bagci O.U., Guldaval F., Muftuoglu C., Mert U., Toz S., Unat D.S., Unat O.S., Polat G., Caner A. (2023). Detection of *Pneumocystis jirovecii* by PCR in patients with lung cancer: A preliminary study. J. Med. Mycol..

[23] Zaini J., A’la Al Maududi A., Fillahihasanah T., Fadhillah M.R., Pradono P., Haryanto B., Adawiyah R., Setianingrum F., Rozaliyani A., Syahruddin E. (2022). *Pneumocystis jirovecii* colonization in bronchoalveolar lavage among naïve non-small cell lung cancer from tertiary respiratory hospital in Jakarta, Indonesia. J. Infect. Dev. Ctries..

[24] Cushion M.T., Tisdale-Macioce N., Sayson S.G., Porollo A. (2021). The persistent challenge of Pneumocystis growth outside the mammalian lung: Past and future approaches. Front. Microbiol..

[25] Brown L., Alanio A., Cruciani M., Barnes R., Donnelly J.P., Loeffler J., Rautemaa-Richardson R., White P.L. (2024). Strengths and limitations of molecular diagnostics for *Pneumocystis jirovecii* pneumonia. Expert Rev. Mol. Diagn..

[26] Schmoldt S., Schuhegger R., Wendler T., Huber I., Söllner H., Hogardt M., Arbogast H., Heesemann J., Bader L., Sing A. (2008). Molecular evidence of nosocomial *Pneumocystis jirovecii* transmission among 16 patients after kidney transplantation. J. Clin. Microbiol..

[27] Singh Y., Mirdha B.R., Guleria R., Kabra S.K., Mohan A., Chaudhry R., Kumar L., Dwivedi S.N., Agarwal S.K. (2019). Genetic polymorphisms associated with treatment failure and mortality in pediatric Pneumocystosis. Sci. Rep..

[28] Liu Y., Fahle G.A., Kovacs J.A. (2018). Inability to culture *Pneumocystis jirovecii*. MBio.

[29] Calderón E.J., De Armas Y., Panizo M.M., Wissmann G. (2013). *Pneumocystis jirovecii* pneumonia in Latin America. A public health problem?. Expert Rev. Anti-Infect. Ther..

[30] Rodríguez Y.D.A., Wissmann G., Müller A., Pederiva M.A., Brum M., Brackmann R., Capó de Paz V., Calderón E.J. (2011). *Pneumocystis jirovecii* pneumonia in developing countries. Parasite J. Société Française Parasitol..

[31] Montazeri A., Mohammadi S., M.Hesari P., Ghaemi M., Riazi H., Sheikhi-Mobarakeh Z. (2023). Preliminary guideline for reporting bibliometric reviews of the biomedical literature (BIBLIO): A minimum requirements. Syst. Rev..

[32] Clarivate™ Plataforma Web of Science 2024. https://clarivate.com/academia-government/scientific-and-academic-research/research-discovery-and-referencing/web-of-science/.

[33] Scopus^®^. https://assets.ctfassets.net/o78em1y1w4i4/28v2L8eQgAGxOnnvZlqJWh/7947feb83982b078ec1d70c297055c34/ELSV_15617_Scopus_Fact_Sheet_Update_WEB.pdf.

[34] Falagas M.E., Pitsouni E.I., Malietzis G.A., Pappas G. (2008). Comparison of PubMed, Scopus, web of science, and Google scholar: Strengths and weaknesses. FASEB J..

[35] Palella F.J., Delaney K.M., Moorman A.C., Loveless M.O., Fuhrer J., Satten G.A., Aschman D.J., Holmberg S.D., the HIV Outpatient Study Investigators (1998). Declining morbidity and mortality among patients with advanced human immunodeficiency virus infection. N. Engl. J. Med..

[36] Mocroft A., Ledergerber B., Katlama C., Kirk O., Reiss P., Monforte A.d.A., Knysz B., Dietrich M., Phillips A., Lundgren J. (2003). Decline in the AIDS and death rates in the EuroSIDA study: An observational study. Lancet.

[37] Kaplan J.E., Benson C., Holmes K.K., Brooks J.T., Pau A., Masur H. (2009). Guidelines for prevention and treatment of opportunistic infections in HIV-infected adults and adolescents. MMWR Recomm. Rep..

[38] Wickramasekaran R.N., Jewell M.P., Sorvillo F., Kuo T. (2017). The changing trends and profile of pneumocystosis mortality in the United States, 1999–2014. Mycoses.

[39] Thomas C.F., Limper A.H. (2004). Pneumocystis pneumonia. N. Engl. J. Med..

[40] Morris A., Wei K., Afshar K., Huang L. (2008). Epidemiology and clinical significance of Pneumocystis colonization. J. Infect. Dis..

[41] Tamburrini E., Mencarini P., Visconti E., Zolfo M., Marinaci S., Zinzi D., Margutti P., Ortona E., Siracusano A. (1998). Potential impact of Pneumocystis genetic diversity on the molecular detection of the parasite in human host. FEMS Immunol. Med. Microbiol..

[42] Flori P., Bellete B., Durand F., Raberin H., Cazorla C., Hafid J., Lucht F., Sung R.T.M. (2004). Comparison between real-time PCR, conventional PCR and different staining techniques for diagnosing *Pneumocystis jiroveci* pneumonia from bronchoalveolar lavage specimens. J. Med. Microbiol..

[43] Helweg-Larsen J. (2004). *Pneumocystis jiroveci*. Applied molecular microbiology, epidemiology and diagnosis. Dan. Med. Bull..

[44] Frenkel J. (1976). *Pneumocystis jiroveci* n. sp. From Man: Morphology, Physiology, and Immunology in Relation to. Natl. Cancer Inst. Monogr..

[45] Calderón E.J., Armas Rodríguez Yd Capó de Paz V. (2011). *Pneumocystis jirovecii*: One hundred years of history. Rev. Cuba. Med. Trop..

[46] Keely S.P., Fischer J.M., Stringer J.R. (2003). Evolution and speciation of Pneumocystis. J. Eukaryot. Microbiol..

[47] Armbruster C., Hassl A., Kriwanek S. (1997). Pneumocystis carinii colonization in the absence of immunosuppression. Scand. J. Infect. Dis..

[48] Llibre J.M., Revollo B., Vanegas S., Lopez-Nuñez J.J., Ornelas A., Marin J.M., Santos J.R., Marte P., Morera M., Zuluaga P. (2013). *Pneumocystis jirovecii* pneumonia in HIV-1-infected patients in the late-HAART era in developed countries. Scand. J. Infect. Dis..

[49] Ekstrand M.L., Shet A., Chandy S., Singh G., Shamsundar R., Madhavan V., Saravanan S., Heylen E., Kumarasamy N. (2011). Suboptimal adherence associated with virological failure and resistance mutations to first-line highly active antiretroviral therapy (HAART) in Bangalore, India. Int. Health.

[50] Amstutz P., Bahr N.C., Snyder K., Shoemaker D.M. (2023). *Pneumocystis jirovecii* infections among COVID-19 patients: A case series and literature review. Open Forum Infect. Dis..

[51] Yu S., Yang T. (2024). Non-HIV Immunocompetent Patient with COVID-19 and Severe *Pneumocystis jirovecii* Pneumonia Co-Infection. Emerg. Infect. Dis..

[52] Watson A.L., Woodford J., Britton S., Gupta R., Whiley D., McCarthy K. (2024). Determining *Pneumocystis jirovecii* colonisation from infection using PCR-based diagnostics in HIV-negative individuals. Diagnostics.

[53] Xue T., Ma Z., Liu F., Du W., He L., Wang J., An C. (2020). *Pneumocystis jirovecii* colonization and its association with pulmonary diseases: A multicenter study based on a modified loop-mediated isothermal amplification assay. BMC Pulm. Med..

[54] Rabacal W., Rayens E., Norris K. (2018). Pneumocystis as a Co-Factor in Pulmonary Diseases. OBM Genet..

[55] Jaramillo Cartagena A., Asowata O.E., Ng D., Babady N.E. (2025). An overview of the laboratory diagnosis of *Pneumocystis jirovecii* pneumonia. J. Clin. Microbiol..

[56] Cederwall S., Ottander E., Björkhem D., Oldberg K., Påhlman L.I. (2025). Performance of Quantitative PCR to Distinguish *Pneumocystis jirovecii* Pneumonia From Colonisation in Immunocompromised Patients. Mycoses.

[57] Costa V.S., Cidade J.P., Medeiros I., Fidalgo P., Moreira H., Miranda T., Póvoa P. (2025). *Pneumocystis jirovecii* Pneumonia Diagnosis with Oropharyngeal Wash PCR in Immunocompromised Patients—A Systematic Review. J. Clin. Med..

[58] Yi J., Wang N., Wu J., Tang Y., Zhang J., Zhu L., Rui X., Guo Y., Xu Y. (2021). Development of a droplet digital polymerase chain reaction for sensitive detection of *Pneumocystis jirovecii* in respiratory tract specimens. Front. Med..

[59] Zhan Y., Gao X., Li S., Si Y., Li Y., Han X. (2022). Development and evaluation of rapid and accurate CRISPR/Cas13-based RNA diagnostics for *Pneumocystis jirovecii* pneumonia. Front. Cell. Infect. Microbiol..

[60] Del Corpo O., Butler-Laporte G., Sheppard D.C., Cheng M.P., McDonald E.G., Lee T.C. (2020). Diagnostic accuracy of serum (1-3)-β-D-glucan for *Pneumocystis jirovecii* pneumonia: A systematic review and meta-analysis. Clin. Microbiol. Infect..

[61] Butler-Laporte G., Smyth E., Amar-Zifkin A., Cheng M.P., McDonald E.G., Lee T.C. (2020). Low-dose TMP-SMX in the treatment of *Pneumocystis jirovecii* pneumonia: A systematic review and meta-analysis. Open Forum Infect. Dis..

[62] Dimeas I.E., Dimeas G.E., Zakynthinos G.E., Tsolaki V. (2025). Individualized Trimethoprim-Sulfamethoxazole Dosing in Non-HIV Patients with Pneumocystis Pneumonia: A Narrative Review of Current Evidence. J. Pers. Med..

[63] Johnson M.D., Lewis R.E., Dodds Ashley E.S., Ostrosky-Zeichner L., Zaoutis T., Thompson G.R., Andes D.R., Walsh T.J., Pappas P.G., A Cornely O. (2020). Core recommendations for antifungal stewardship: A statement of the mycoses study group education and research consortium. J. Infect. Dis..

[64] Gavriilaki E., Evangelidis P., Kotsiou N., Sakellari I., Cornely O.A., Pagano L., Salmanton-García J. (2025). Antifungal prescription and stewardship in hematology and hematopoietic stem cell transplantation units worldwide: An international survey of EHA-SWG Infections in Hematology. Bone Marrow Transplant..

[65] Haensel L., Sprute R., Grothe J., Robert-Gangneux F., Gangneux J.-P., Meis J.F., Cornely O.A., Koehler P. (2025). The 2025 EQUAL Pneumocystis Score—An ECMM tool to measure QUALity in Pneumocystis pneumonia management. JAC-Antimicrob. Resist..

[66] Alanio A., Hauser P.M., Lagrou K., Melchers W.J., Helweg-Larsen J., Matos O., Cesaro S., Maschmeyer G., Einsele H., Donnelly J.P. (2016). ECIL guidelines for the diagnosis of *Pneumocystis jirovecii* pneumonia in patients with haematological malignancies and stem cell transplant recipients. J. Antimicrob. Chemother..

[67] Alegria W., Patel P.K. (2021). The current state of antifungal stewardship in immunocompromised populations. J. Fungi.

[68] Fishman J.A., Gans H., on behalf of the AST Infectious Diseases Community of Practice (2019). *Pneumocystis jiroveci* in solid organ transplantation: Guidelines from the American Society of Transplantation Infectious Diseases Community of Practice. Clin. Transplant..

[69] Rivero L., de la Horra C., Montes-Cano M.A., Rodríguez-Herrera A., Respaldiza N., Friaza V., Morilla R., Gutiérrez S., Varela J.M., Medrano F.J. (2008). *Pneumocystis jirovecii* transmission from immunocompetent carriers to infant. Emerg. Infect. Dis..

[70] Chen Y., Xu X., Huang Z., Lai X., Li C., Chen J., Wu W., Chipusu K., Zeng Y. (2025). Diagnostic predictive evaluation of pneumocystis jirovecii pneumonia using digital chest CT analysis combined with clinical features. Front. Physiol..

[71] Lian Q., Song X., Yang J., Wang L., Xu P., Wang X., Xu X., He J., Ju C. (2024). Alterations of lung microbiota in lung transplant recipients with *pneumocystis jirovecii* pneumonia. Respir. Res..

