# Supplementary material for: Past Achievements, Present Gaps, and Future Priorities in Pneumocystis jirovecii Research: A Global Bibliometric Analysis
Source: Pathogens. 2026 May 14;15(5):530. doi: 10.3390/pathogens15050530 (PMC13209675; doi:10.3390/pathogens15050530)
Supplement: Supplementary file 1 [file pathogens-15-00530-s001.zip › Supplementary Material S1.pdf]

The BIBLIO checklist for reporting the bibliometric reviews of the biomedical literature

Past Achievements, Present Gaps, and Future Priorities in *Pneumocystis jirovecii* Research: A Global Bibliometric Analysis

Bryan Ortiz [1,†], Jonathan Muñoz-Tabora [2,†], Kateryn Aguilar [1], Gustavo Fontecha [1], Gabriela Matamoros [1], Lelany Pineda-Garcia [3], Nancy Alvarez-Corrales [3], Jaime Palomares-Marín [4], Claudia L. Cueto-Aragón [5], Yaxsier de Armas [4,6,*] and Enrique J. Calderón [7,8,*]

[1] Instituto de Investigaciones en Microbiología, Facultad de Ciencias, Universidad Nacional Autónoma de Honduras, Tegucigalpa 11101, Honduras; bryan.ortiz@unah.edu.hn (B.O.); kateryn.aguilar@unah.edu.hn (K.A.); gustavo.fontecha@unah.edu.hn (G.F.); gabriela.matamoros@unah.edu.hn (G.M.)

[2] Electric Engineering Department, National Autonomous University of Honduras, Tegucigalpa 04001, Honduras; jonathan.munoz@unah.edu.hn

[3] Departamento de Microbiología, Escuela de Microbiología, Facultad de Ciencias, Universidad Nacional Autónoma de Honduras, Tegucigalpa 11101, Honduras; lelany.pineda@unah.edu.hn (L.P.-G.); nancy.alvarez@unah.edu.hn (N.A.-C.)

[4] Departamento de Microbiología y Patología, Centro Universitario de Ciencias de la Salud, Universidad de Guadalajara, Guadalajara 44100, Mexico; jaime.palomares@academicos.udg.mx

[5] Hospital Infantil "Eva Sámano de López Mateos", Morelia 58253, Mexico; claudiacueto22@gmail.com

[6] Pathology Department, Hospital Center, Institute of Tropical Medicine "Pedro Kourí", Havana 11400, Cuba

[7] Instituto de Biomedicina de Sevilla, Hospital Universitario Virgen del Rocío, Consejo Superior de Investigaciones Científicas/Universidad de Sevilla, 41013 Seville, Spain

[8] Centro de Investigación Biomédica en Red de Epidemiología y Salud Pública, 28029 Madrid, Spain

* Correspondence: yaxsier.dearmas@academicos.udg.mx (Y.d.A.), ecalderon@us.es (E.J.C.)

† These authors contributed equally to this work.

Section/Topic	Item No.	Checklist item	Reported on page No.
**Title**			
Identification	1	Identify the report as a bibliometric review in the title.	Reported on page No. 1
Issues/topics	2	Indicate the key issues/topics under investigation and coverage of time period.	Reported on page No. 1
**Abstract**			
Structured summary	3	Structured summary including (as applicable): background, methods, results (key findings) and conclusions.	Partially reported, p. 1. The abstract includes the recommended components; however, according to the journal's format, it was presented as an unstructured abstract without separate headings.
**Introduction/ Background**			
Justification/ Rationale/ Explanation	4	Present review of existing knowledge and epidemiological information.	Reported on page No. 2-4
Objectives	5	Statement of the objective (s) or question (s).	Reported on page No. 4
**Methods**			
Search engines (data sources)	6	Describe all information sources (such as electronic databases, contact with study authors, trial registers or other grey literature sources).	Reported on page No. 4
Search strategy	7	Keywords and systematization criteria (date of search, language, type of document) for the search.	Reported on page No. 5
Time period	8	The period that the review covers and the justification.	Reported on page No. 5

Eligibility criteria	9	Describe all inclusion and exclusion criteria; languages; study design, type of publication and time period.	Reported on page No. 4-6
Data refinement (data selection procedure)	10	Remove the irrelevant articles; inspection to eliminate duplicate and unrelated articles (after evaluation of the title, abstract and content).	Reported on page No. 6
Quality assessment (optional)	11	Assessment of papers by three authors and the use of assessing checklists.	N/A
Data synthesis	12	Describe the methods used for summarizing, handling, synthesis, tabulations or schematic displays. Describe how the data were analysed.	Reported on page No. 4-6
**Results**			
Descriptive findings (statistics)	13	- Provide details of the search and selection process in a flow diagram.<br>- Number of citations retrieved (number of publication, year of publication, type of documents, country of publication, articles with the highest impact, most impactful authors, most impactful articles, authors with the highest production, top journals, top institutions, …)	Reported on page No.7-21
Schematic map and trend	14	Summarize and/or present the schematic maps and trends using an appropriate software to present citations, journals, authors, top journals, time trends, emerging literature, and any relevant indicators (as applicable) [1-5].	Reported on page No.7-21
Tabulation and summarizing the findings	15	General recommendation: Studies under consideration could be summarized and organized by different subtitles and different scenarios. Regardless, results need to be presented in separate tables covering each subtitle. The followings are some options that could help to summarize the findings.<br><br>*Option 1:*<br>- Start the presentation with a historical view [when and who first published on the topic].<br>- Report on review papers. The result should be listed in a separate table. Also, specify the review type (scoping review, narrative review, systematic review, and meta-analysis).<br>- Summarize the findings according to the study designs and main study types.<br>*Option 2:*	Reported on page No.7-21

		- Start the presentation with a historical view [when and who first published on the topic].<br>- Report on review papers. The result should be listed in a separate table. Also, indicate the review type (scoping review, narrative review, systematic review, and meta-analysis) should be specified.<br>- Summarize the findings according to outcome measures or populations. For example, see [6].<br>*Option 3:*<br>- Start the presentation with a historical view [when and who first published on the topic].<br>- Report on review papers. The result should be listed in a separate table. Also, specify the review type (scoping review, narrative review, systematic review, and meta-analysis).<br>- Summarize the findings according to concept [7].<br>*Option 4.*<br>- Start the presentation with a historical view [when and who first published on the topic].<br>- Report on review papers. The result should be listed in a separate table, and also specify the review type (scoping review, narrative review, systematic review, and meta-analysis).<br>- Summarize the findings according to different subtitles relevant to the main topic [8].	
Synthesis of findings	16	Synthesize the findings as much as possible, find the gap, and propose a model, hypothesis, etc. (if applicable).	Reported on page No. 24-27
**Discussion**			
Summary of evidence	17	Summarize the main findings. The findings should be presented in more "general" or "accessible" terms.	Reported on page No. 24
Interpretation	18	Include interpretation consistent with results. Explanations for observed outcomes, similarities, and differences reported would be essential.	Reported on page No. 24-28
Strengths and limitations	19	Discuss the strengths and limitations.	Reported on page No. 29
Conclusion(s)	20	Provide a general interpretation of the results with respect to the review questions and objectives, as well as potential implications.	Reported on page No. 30

1. McDougal L, Dehingia N, Cheung WW, Dixit A, Raj A. COVID-19 burden, author affiliation and women's well-being: A bibliometric analysis of COVID-19 related publications including focus on low-and middle-income countries. eClinicalMedicine 2022; 52: 101606.
2. Henstock L, Wong R, Tsuchiya A, Spencer A. Behavioral theories that have influenced the way health state preferences are elicited and interpreted: A bibliometric mapping analysis of the ttime trade-off method with VOSviewer visualization. Front Health Serv 2022; 2: 848087.

3. Bodea F, Bungau SG, Negru AP, Radu A, Tarce AG, Tit DM, et al. Exploring new therapeutic avenues for ophthalmic disorders: Glaucoma-related molecular docking evaluation and bibliometric analysis for improved management of ocular diseases. Bioengineering 2023; 10(8): 983.
4. Sang XZ, Wang CQ, Chen W, Rong H, Hou LJ. An exhaustive analysis of post-traumatic brain injury dementia using bibliometric methodologies. Front Neurol 2023; 14: 1165059.
5. Ramli MI, Hamzaid NA, Engkasan JP, Usman J. Respiratory muscle training: a bibliometric analysis of 60 years' multidisciplinary journey. Biomed Eng Online 2023; 22(1): 50.
6. Akosman I, Kumar N, Mortenson R, Lans A, De La Garza Ramos R, Eleswarapu A,et al. Racial differences in perioperative complications, readmissions, and mortalities after elective spine surgery in the United States: A systematic review using AI-assisted bibliometric analysis. Glob Spine J 2023: 21925682231186759.
7. Tavousi M, Mohammadi S, Sadighi J, Zarei F, Kermani RM, Rostami R, Montazeri A. Measuring health literacy: A systematic review and bibliometric analysis of instruments from 1993 to 2021. Plos One 2022; 17(7): e0271524.
8. Montazeri A. Health-related quality of life in breast cancer patients: A bibliographic review of the literature from 1974 to 2007. J Exp Clin Cancer Res 2008; 27: 32.

Rights and permissions: The original source of the checklist is: Montazeri A, Mohammadi S, M.Hesari P, Ghaemi M, Riazi H, Sheikhi-Mobarakeh Z. Preliminary guideline for reporting bibliometric reviews of the biomedical literature (BIBLIO): a minimum requirements. *Systematic Reviews* 2023; 12: 239. doi.org/10.1186/s13643-023-02410-2 The article is licensed under a Creative Commons Attribution 4.0 International License (http://creativecommons.org/licenses/by/4.0/). A changes was made to the original checklist to add in full references to the cited sources.
