# Supplementary material for: Past Achievements, Present Gaps, and Future Priorities in Pneumocystis jirovecii Research: A Global Bibliometric Analysis
Source: Pathogens. 2026 May 14;15(5):530. doi: 10.3390/pathogens15050530 (PMC13209675; doi:10.3390/pathogens15050530)
Supplement: Supplementary file 1 [file pathogens-15-00530-s001.zip › Supplementary Material S3.pdf]

The following search strategies were applied:

Streptococcus pneumoniae:

TITLE-ABS-KEY (pneumonia AND "Streptococcus pneumoniae")

Haemophilus influenzae:

TITLE-ABS-KEY (pneumonia AND "Haemophilus influenzae")

Staphylococcus aureus:

TITLE-ABS-KEY (pneumonia AND "Staphylococcus aureus")

Klebsiella pneumoniae:

TITLE-ABS-KEY (pneumonia AND "Klebsiella pneumoniae")

Mycoplasma pneumoniae:

TITLE-ABS-KEY (pneumonia AND "Mycoplasma pneumoniae")

Influenza virus:

TITLE-ABS-KEY (pneumonia AND influenza)

SARS-CoV-2 / COVID-19:

TITLE-ABS-KEY (pneumonia AND ("SARS-CoV-2" OR "COVID-19"))

Respiratory Syncytial Virus (RSV):

TITLE-ABS-KEY (pneumonia AND ("respiratory syncytial virus" OR RSV))

Pneumocystis jirovecii / Pneumocystis pneumonia (PCP):

TITLE-ABS-KEY (pneumonia AND ("Pneumocystis jirovecii" OR Pneumocystis OR PCP))

Aspergillus spp.:

TITLE-ABS-KEY (pneumonia AND Aspergillus)
